# Cost-effectiveness of a mTB-Tobacco intervention for smoking cessation in people with tuberculosis: an economic evaluation of a cluster randomised controlled trial

**DOI:** 10.1016/j.lansea.2026.100776

**Published:** 2026-05-05

**Authors:** Jinshuo Li, Steve Parrott, Maham Zahid, Fahmidur Rahman, Mahmoud Danaee, Shakhawat Hossain Rana, Asiful Chowdhury, Saeed Ansaari, Ai Keow Lim, Melanie Boeckmann, Amina Khan, Rumana Huque, John Norrie, Kamran Siddiqi

**Affiliations:** a Department of Health Sciences, University of York, Heslington, York, YO10 5DD, UK; b The Initiative, Orange Grove Farm, Main Korung Road, Banigala, Islamabad, 44000, Pakistan; c ARK Foundation, Suite A-1, C-3 & C-4, House # 06, Road # 109, Gulshan-2, Dhaka, 1212, Bangladesh; d Department of Social and Preventive Medicine, Faculty of Medicine, University of Malaya, 50603, Kuala Lumpur, Malaysia; e Usher Institute, Usher Building, University of Edinburgh, 5-7 Little France Road, Edinburgh BioQuarter – Gate 3, Edinburgh, EH16 4UX, UK; f Department of Global Health, Institute of Public Health and Nursing Research IPP, University of Bremen, Universitaetsallee 1B, 28359, Bremen, Germany; g Centre for Public Health, Institute of Clinical Sciences, Royal Victoria Hospital, Queen's University, Belfast, BT12 6BA, UK; h Hull York Medical School, University of York, York, YO31 0TN, UK

**Keywords:** Cost-effectiveness, Tuberculosis, Smoking cessation, Mobile health, Economic evaluation, Randomised controlled trial, Low- and middle-income countries

## Abstract

**Background:**

High prevalence of smoking tobacco among people with tuberculosis (TB) contribute towards poor outcomes in low- and middle-income countries. A mobile phone-based intervention for smoking cessation among this population (mTB-Tobacco) was evaluated for its cost-effectiveness alongside a cluster randomised controlled trial in Pakistan and Bangladesh.

**Methods:**

A two-arm superiority cluster randomised controlled trial with 6 months follow up was conducted between September 2023 and January 2025 in Dhaka, Bangladesh and Punjab, Pakistan. The trial compared the mTB-Tobacco intervention with usual care as control. Participants included those older than or equal to 15 years of age, diagnosed with drug-sensitive pulmonary TB in the past 4 weeks, smoked tobacco daily but willing to quit, and had access to mobile phones. Eighteen TB health facilities (cluster) were randomised to mTB-Tobacco group (n = 720 participants) and nine to usual care (n = 360 participants). The primary analysis was an incremental cost-utility analysis from a public/voluntary sector perspective and primary outcome measure was Quality-Adjusted Life Years (QALYs). Total costs included the costs of TB treatment, costs of intervention or control, and costs of doctor visit and hospital stay. Secondary and sensitivity analyses were also conducted.

**Findings:**

Total costs were INT$ (international dollars) 36.17 (95% CI 3.65–65.81) higher and QALYs were 0.017 (95% CI 0.003–0.030) higher in mTB-Tobacco group than usual care group. Incremental cost-effectiveness ratio was calculated at INT$2127.64 per QALY gained. Estimates by country suggested mTB-Tobacco being unlikely cost-effective in Bangladesh (ICER = INT$4261.11 per QALY gained) but likely cost-effective in Pakistan (ICER = INT$1024.29 per QALY gained).

**Interpretation:**

If decision makers in the public/voluntary sector are willing to pay over INT$2100 for one additional QALY gained, mTB-Tobacco intervention could likely be cost-effective.

**Funding:**

The UK NIHR Global Health Research Unit on Respiratory Health (RESPIRE) (NIHR132826).

**Research in context**

**Evidence before this study**

Tobacco use increases the risk of death during tuberculosis (TB) treatment, and the risk of recurrence or relapse after TB treatment. However, smoking cessation often goes unaddressed among people who were diagnosed with TB. We searched MEDLINE, PubMed, and the Cochrane Library (Systematic Reviews and Trials) from Jan 1, 1980, to Mar 13, 2026, for studies assessing the cost-effectiveness of smoking cessation in people with TB and smoke tobacco. Keywords used in the search were ‘‘tuberculosis’’ AND ‘‘smoking cessation’’ AND “cost-effectiveness” without language restrictions. We identified one model-based study based on Canadian Inuit community, and two trial-based studies. The trial in South Africa found no clinical effectiveness of brief motivational interview and follow-up text messages on reducing hazardous drinking and/or smoking cessation among people with TB. The other trial in Bangladesh and Pakistan found cytisine plus brief behavioural support not effective comparing to brief behavioural support in smoking cessation. Neither could conclude cost-effective because of the clinical ineffectiveness. However, both reported a considerably higher costs with minimal difference in quality-adjusted life years (QALYs). Given the high burden of TB and constrained resources in the low- and middle-income countries (LMICs), evidence on economic evaluation of tobacco cessation interventions are needed for informing policy changes.

**Added value of this study**

Our findings show that mTB-Tobacco, a mobile phone based smoking cessation intervention, among people with TB who smoked tobacco, costed much more than usual care despite yielding sufficient effectiveness both in terms of abstinence and quality of life. The resulting incremental cost-effectiveness ratio (ICER) was high relative to the local circumstances. This contradicts the common narrative that digital health interventions provide a universally low-cost pathway to scalable and sustainable outcomes. The mTB-Tobacco intervention showed potential of reducing participants’ healthcare expenditure, which corresponded with the improved quality of life. But it was also associated with increased lost income due to higher number of absent days from work. Country-specific results illustrate the difference between the two countries. The ICER in Bangladesh was much higher than that in Pakistan and far higher than the estimated willingness-to-pay threshold in Bangladesh. In contrast, the ICER in Pakistan makes the intervention likely to be cost-effective locally.

**Implications of all the available evidence**

Our findings suggested that the mTB-Tobacco intervention was effective but at a much higher cost over a 6-month follow-up. Long-term evaluation beyond 6 months is needed to provide a more comprehensive assessment for a smoking cessation intervention. Though proven to be effective in high income countries, adoption in LMICs require localised evaluations both in clinical outcomes and cost-effectiveness. In countries like Bangladesh and Pakistan, digital health interventions must produce considerably more benefits to justify its additional costs relative to traditional in-person interventions. These interventions must be carefully evaluated against low-tech alternatives in the local settings to ensure their scalability and sustainability.

## Introduction

Over 20% of people with tuberculosis (TB) in Bangladesh and Pakistan are currently smoking tobacco.^1^ Around 10% of new and relapse TB in both countries were attributed to smoking.^2^^,^^3^ Evidence suggested that tobacco use increased the likelihood of mortality during treatment among people with TB and was shown to be a risk factor for other unfavourable treatment outcomes, including recurrence or relapse and treatment failure.^4^

Diagnosis of TB could be a motivation for people to quit smoking as they could be more receptive to the suggestion and benefit from quitting. However, in Bangladesh and Pakistan, availability of support for smoking cessation is limited and the costs of available interventions are partially covered at best.^5^ This, combined with the fact that the importance of smoking cessation among people diagnosed with TB is often overlooked,^6^ poses an obstacle for people with TB who smoke tobacco but want to quit.

To provide technical guidance on tobacco cessation that can be used by member states, the World Health Organization (WHO) made conditional recommendations of digital tobacco cessation interventions with moderate-certainty evidence on automated text-messaging interventions, low-certainty evidence on smartphone apps and artificial intelligence-based interventions, and very low-certainty evidence on internet-based interventions.^7^ Whilst automated text-messaging interventions are recommended as one of the six types of cost-effective and affordable tobacco cessation interventions, the only cost-effectiveness analysis identified was undertaken in the United Kingdom (UK) and the recommendation was based on the WHO CHOICE analysis of population-wide impact.^7^ There is a lack of trial- or implementation-based evidence in low- and middle-income countries (LMICs) to corroborate this recommendation.

A text-messaging intervention was previously developed by the investigators in collaboration with the WHO Be Healthy Be Mobile team and made available in the public domain.^8^ We adapted this intervention for the current study, and it was reviewed, translated, and tested by patients and public involvement consultations and a pilot study, before a multi-centre, superiority, cluster randomised controlled trial (cRCT) was conducted.^9^ This trial, with a 6-month follow-up period, was to assess the mTB-Tobacco intervention over and above usual care in people with drug-sensitive TB who smoke tobacco daily in achieving biochemically verified continuous abstinence. We found that the mTB-Tobacco intervention was effective in helping participants achieve continuous abstinence at the end of follow-up (adjusted relative risk = 3.12; 95% CI 1.9–6.5).^10^ The present study reports the results of cost effectiveness analysis of mTB-Tobacco intervention over and above usual care, from a public/voluntary sector perspective over 6 months, conducted as a part of the cRCT.

## Methods

The cRCT was conducted in Dhaka, Bangladesh and Punjab, Pakistan from September 2023 to January 2025.^10^ Twenty-seven TB clinics (clusters) were randomised in 2:1 ratio to mTB-Tobacco (18 clinics) or usual care (9 clinics) groups. Fifteen clinics were in Bangladesh (10 in mTB-Tobacco group, 5 in usual care group). Twelve clinics were in Pakistan (8 in mTB-Tobacco group, 4 in usual care group).

The mTB-Tobacco intervention consisted of a series of text messages plus one education leaflet for TB. An automated digital messaging system was set up to send the messages to participants according to a pre-specified schedule. The messages contain contents of behavioural changes to quit tobacco use and motivational and informative messages for TB treatment. Each participant was scheduled to receive 134 messages over the 6 months period. In the first two months, the frequency of messages would be 4 to 5 messages per day, in next two months the frequency would reduce to 1 to 2 messages per day and in the last two months there would be 1 message sent per week.

The control group (usual care) received one leaflet containing information about the harmful effects of tobacco and advice on stopping its use, in addition to the same education leaflet for TB received by intervention group.

Eligible TB clinics were government approved, and registering at least 50 new TB patients a month. Eligible patients were at least 15 years old (considered as adult TB patients in Bangladesh and Pakistan), diagnosed with drug sensitive pulmonary TB in the last four weeks, currently smoking tobacco daily but willing to quit, having access to mobile phones, and willing and able to provide written consent. Patients who were receiving retreatment for TB, treatment for multidrug resistance, miliary or extrapulmonary TB, or treatment for tobacco dependence were excluded. This study is registered as an International Standard Randomised Controlled Trial, number ISRCTN 861971818. For further details of the trial methods, please refer to the published protocol.^9^

As the trial was conducted in Bangladesh and Pakistan, the monetary components were collected in local currency and converted into international dollars (INT$) before being combined and presented. All monetary values are presented in INT$ 2024 price (Bangladeshi Taka [BDT] 29.578 = Pakistani Rupee [PKR] 66.928 = INT$1.00).^11^

The costs of messages sent and technical support for the system of mTB-Tobacco were recorded by the third-party which was contracted to provide short message services and allocated evenly to each participant in the mTB-Tobacco group. The leaflets given out in both groups were valued by their printing costs, which were INT$0.61 per leaflet in Bangladesh and INT$0.37 per leaflet in Pakistan. The study team confirmed if leaflets were given as scheduled.

TB treatment consisted of anti-TB medicines, sputum tests and visits to TB clinics. The completion of anti-TB medicines was recorded in the routine TB treatment card, relevant information of which was extracted at the end of treatment. The number of sputum tests taken by participants was extracted from TB treatment card at baseline, 9-week and 6-month follow-ups. The number of visits to TB clinics was collected by self-reported Case Report Forms (CRFs) at baseline and 6-month follow-up, along with number of visits to doctors other than in TB clinics, number of days in hospital, participants’ spending on fees paid to public or voluntary services, private services, travelling expenses to receive care, and spending on tobacco. The unit costs of components of TB treatment and wider healthcare were estimated by country using published literature, inflated to 2024 and converted to INT$ (Supplementary Material 1 Supplementary Table S1).

Participants' spending on care was reported in monetary terms at baseline and 6 months. Participants’ lost income was estimated by applying average income to the number of days off work which was collected at baseline and 6 months. The average income by sex and occupational categories was extracted from Labour Force Survey in both countries,^12^^,^^13^ then inflated and converted to INT$ 2024 (Supplementary Material 1 Supplementary Table S2).^11^^,^^14^ These were applied to each participant by their sex and occupation reported if in employment at baseline. If participants reported not in employment at baseline but had a job at 6 months, overall average income of all occupational categories was applied. If they did not have a job at the time or did not take any days off work, they were considered not having lost any income.

EQ-5D-5L^15^ was collected as part of the CRFs at baseline and 6 months. It is a generic preference-based measure of health-related quality of life consisting of 5 domains, each with 5 severity levels, and a visual analogue scale (VAS). A complete set of responses of the 5 domains was converted to a utility value using a population tariff, with death anchors at 0. The utility values at multiple timepoints were used to derive quality-adjusted life years (QALYs) using area under the curve approach.^16^ To account for death occurred in the study, days in the study was calculated using the date of death or date of completing 6 months CRF minus the date of baseline and used to construct the time horizon of the curve. Due to the lack of population tariff for either country in the study, an Indian value set was used to generate utilities.^17^

Biochemically verified continuous abstinence at 6 months post randomisation was the primary outcome.^18^ Abstinence was defined as self-report of not having used more than 5 cigarettes, bidis, water pipe sessions since the quit date (5 days after enrolment), verified biochemically by a breath carbon monoxide (CO) reading of less than 10 ppm at month 6, or in the case of concomitant smokeless tobacco use, by a urine cotinine (a nicotine metabolite) level <3 on a Accutest® NicAlertTM strip (equivalent to 100–200 ng/mL cotinine).^9^ Participants who were lost to follow-up or failed to take biochemical verification, were considered as continued smokers. Following Russell standard,^19^ those who died were excluded when estimating abstinence.

### Statistical analysis

Missing data level was examined for all measures at all time points by groups and in total. Missing data were handled based on the framework outlined by Faria et al.^20^ Missing values of baseline data were rare and unrelated to the group allocation, therefore imputed by the mean of the measure of the pooled sample of both groups.^21^ Missing values of measures at follow-ups were handled using multiple imputation with chained equation method, following Rubin's rule and assuming missing at random (MAR).^22^ After examination of missing data (Supplementary Material 2), age, sex, country, site, occupation, days in the study, death status, biochemically verified continuous abstinence status at 6 months, costs of anti-TB medicines, costs of sputum tests at all timepoints, costs and spending of visits to TB clinics, and doctor and hospital stays, spending on tobacco, lost income, and EQ-5D-5L utility and VAS at baseline and 6 months were included in the imputation model. The highest percentage of the missing data was 13%, the number of imputations was therefore set as 13.^21^ The imputation was performed by allocation group.

All analyses were conducted on the imputed dataset, unless otherwise specified. Participants were analysed in their randomised groups. Analyses were outlined in the protocol^9^ and performed in StataNow MP 19. No discounting was applied as the time horizon did not exceed one year.

The primary analysis was an incremental cost-utility analysis from a public/voluntary sector perspective over 6 months. The public/voluntary sector perspective restricted the costs included to those incurred in healthcare services that was funded by government or voluntary organisation. The total costs therefore included costs of mTB-Tobacco and usual care, costs of TB treatment, and costs of doctor visits and hospital stays. The effect measure was QALYs. The incremental costs and QALYs were estimated using generalised linear regression, with covariates that showed a significance (coefficient P value < 0.05) as fixed effects and site (cluster) as random intercepts. An incremental cost-effectiveness ratio (ICER) was then calculated by dividing the incremental costs by the incremental QALYs, when both were positive.

The uncertainty was examined using a non-parametric bootstrap technique.^23^ Five thousand replicated samples were drawn with site as cluster and incremental costs and QALYs were estimated after multiple imputation, with the same model (number of imputation = 5), of each of them. The 5000 iterations were first used to construct 95% confidence interval (CI) for incremental costs and QALYs respectively. They were then plotted onto a cost-effectiveness plane (CEP) to demonstrate jointly the uncertainty surrounding ICER. Finally, they were used to construct cost-effectiveness acceptability curve (CEAC) to illustrate the probability of cost-effectiveness at various thresholds of willingness to pay (WTP).^24^ Since no official WTP was published in either country at the time of analysis, estimated WTPs based on price elasticity were inflated, converted and adopted.^11^^,^^14^^,^^25^ The estimated WTP in Bangladesh ranged from INT$163.14 per QALY to INT$2321.96 per QALY and in Pakistan from INT$386.56 per QALY to INT$2972.54 per QALY.

Secondary analyses included an incremental cost-effectiveness analysis of treatment costs with abstinence at 6 months, and incremental cost analyses of participants' spending on care and lost income. The former was carried out by dividing the mean mTB-Tobacco and usual care costs per participant by the respective biochemically verified continuous abstinence rate at 6 months to calculate costs of intervention and control per abstainer. Incremental costs per additional abstainer were calculated by dividing the difference between the costs of intervention and control by the difference in abstinence rate between groups. This was to assess the direct effect of the intervention and control on smoking cessation. Incremental cost analyses were carried out for participants’ spending on care and for participants lost income. It should be noted that unlike spending on care that was reported by participants, loss of income was estimated based on limited information, which might entail higher uncertainty.

Sensitivity analyses included complete case analysis and scenario-based missing not at random (MNAR) analysis. A set of complete case analyses were carried out following the methods of the primary and secondary analyses to assess the impact of missing data and death during follow-up. These were performed on those who had complete data on all variables included in the analysis and excluded those who died. To examine the robustness of the MAR assumption, MNAR analyses were carried out using pattern mixture modelling.^20^ This method assumes that data are MNAR and sets out scenarios for imputing to reflect this assumption. In the current study, we assumed that those who had missing values at follow-ups either need more healthcare services (higher costs) or experience worse health (lower utility values), or both at the same time. To examine how these assumptions affected the results under MAR assumption, the incremental costs and QALYs were re-estimated based on data with 1) imputed costs were increased by 20%, 40% and 60%; 2) imputed EQ-5D-5L utility values were reduced by 20%, 40% and 60%; 3) the combination of 1) and 2).

Country-specific analyses following the methods described above were added as a post-hoc analysis.

### Ethics statement

This study was performed in accordance with the Declaration of Helsinki. Ethical approval was granted by Edinburgh Medical School Research Ethics Committee (reference number: EMREC-RESPIRE-23-01), Bangladesh Medical Research Council (reference number: BMRC/NREC/2022-2025/176) and the Pakistan National Institutes of Health, National Bioethics Committee for Research (reference number: No.4-87/NBC-929/23/1873). Written informed consent was obtained from all participants prior to taking part in the research.

### Role of the funding source

The NIHR had no role in the design and conduct of the study; collection, management, analysis, and interpretation of the data; preparation, review, or approval of the manuscript; and decision to submit the manuscript for publication.

## Results

In total, 720 participants were randomised to the mTB-Tobacco group and 360 randomised to the usual care group. No significant difference was found between groups in age and sex. The difference in CRF return rate between groups was significant because the recorded deaths were significantly higher in the usual care group than in the mTB-Tobacco group (Table 1).

**Table 1:** Baseline characteristics and CRF return rate at 6 months, by group.

	mTB-Tobacco	Usual care	P-value^a^
Bangladesh	**n**=**400**	**n**=**200**	
Age, mean (SD)	46.7 (15.5)	47.8 (15.5)	0.423
Male, n (%)	399 (100%)	200 (100%)	0.479
Recorded deaths during follow-up, n (%)	8 (2%)	9 (5%)	0.082
CRF return at 6 months, n (%)	381 (95%)	185 (93%)	0.170
Pakistan	**n**=**320**	**n**=**160**	
Age, mean (SD)	51.1 (15.8)	49.9 (16.4)	0.432
Male, n (%)	299 (93%)	145 (91%)	0.270
Recorded deaths during follow-up, n (%)	17 (5%)	18 (11%)	**0.018**
CRF return at 6 months, n (%)	286 (89%)	133 (83%)	0.053
All participants	**n**=**720**	**n**=**360**	
Age, mean (SD)	48.7 (15.8)	48.8 (15.9)	0.957
Male, n (%)	698 (97%)	345 (96%)	0.344
Recorded deaths during follow-up, n (%)	25 (3.5%)	27 (7.5%)	**0.004**
CRF return at 6 months, n (%)	667 (93%)	318 (88%)	**0.019**

a P-value in bold indicates significant (<0.05).

The adaptation of the mTB-Tobacco intervention to the local contexts costed INT$20,544.47 in total. This was considered ‘sunk costs’ and should not contribute to decision making, so it was not included in the following analysis. Nearly 40% of the mTB-Tobacco intervention delivery costs were attributed to supporting staff and the remaining was accounted for by messaging service (Table 2). The mean intervention costs were INT$38.78 (SD 0.12) per participant in the mTB-Tobacco group. The mean control costs were INT$1.01 (SD 0.23) per participant in the usual care group. Detailed summary of TB treatment and healthcare costs, spendings on care, lost income and EQ-5D-5L were presented in Supplementary Material 3.

**Table 2:** Costs of mTB-Tobacco and usual care.

Costs, INT$	mTB-Tobacco (n = 720)	Usual care (n = 360)
mTB-Tobacco intervention delivery		
Total supporting staff for mTB-Tobacco	10,459.00	–
Total messages	17,099.56	–
*Per participant, mean*	*38.28*	-
Education leaflets		
*Per participant, mean (SD)*	*0.50 (0.12)*	*1.01 (0.23)*
Total treatment costs per participant, mean (SD)	**38.78 (0.12)**	**1.01 (0.23)**

**Fig. 1:**
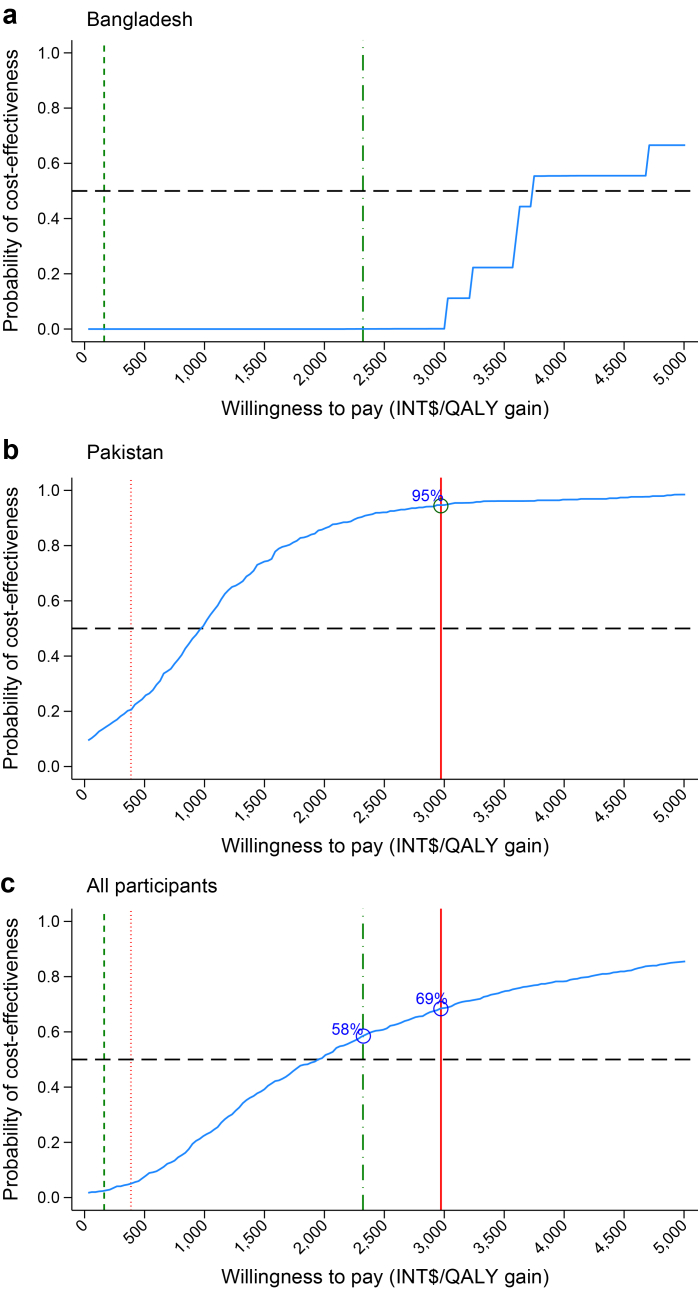
**Cost-effectiveness acceptability curve (CEAC) showing the probability of mTB-Tobacco being cost-effective compared to usual care based on the primary analysis (n = 1080)**. Panel a for Bangladesh. Panel b for Pakistan. Panel c for all participants. Reference lines in the figure show the estimated willingness to pay (WTP) threshold for Bangladesh and Pakistan: Green short-dash line = Bangladesh WTP lower boundary INT$163.14/QALY gained; Red tight dots line = Pakistan WTP lower boundary INT$386.56/QALY gained; Green dash-dot line = Bangladesh WTP upper boundary INT$2321.96/QALY gained; Red solid line = Pakistan WTP upper boundary INT$2972.54/QALY gained.

After multiple imputation of missing data, the ICER estimated in the primary analysis found that the mTB-Tobacco costed INT$36.17 (95% CI 3.65–65.81) more than the usual care and yielded additional 0.017 QALYs (95% CI 0.003–0.030), with an ICER of INT$2127.64 per QALY gain from a public/voluntary sector perspective (Table 3). The probability of mTB-Tobacco being cost-effective remained below 50% for WTP thresholds up to approximately INT$2000 (Fig. 1). Beyond this point, the probability increased, reaching 58% and 69% at the maximum thresholds for Bangladesh and Pakistan, respectively. The CEP were shown in Supplementary Material 4 Supplementary Figure S1.

**Table 3:** Primary analysis: Incremental cost-utility analysis from the public/voluntary sector perspective.

	Bangladesh		Pakistan		All participants	
	mTB-Tobacco (n = 400)	Usual care (n = 200)	mTB-Tobacco (n = 320)	Usual care (n = 160)	mTB-Tobacco (n = 720)	Usual care (n = 360)
Costs in the 6 months before baseline, Mean (SE), INT$						
Sputum test	1.39 (0.03)	1.48 (0.04)	5.91 (0.05)	5.78 (0.00)	3.40 (0.09)	3.39 (0.11)
TB clinic	0.30 (0.03)	0.11 (0.03)	0.07 (0.03)	0.17 (0.08)	0.20 (0.02)	0.14 (0.04)
Doctor & hospital stay	7.78 (1.67)	11.79 (2.38)	45.84 (14.15)	51.90 (12.57)	24.70 (6.39)	29.62 (5.83)
Total	9.47 (1.67)	13.39 (2.39)	51.82 (14.15)	57.85 (12.57)	28.29 (6.40)	33.15 (5.85)
Costs from baseline to 6 months, Mean (SE), INT$						
Intervention/control	38.89 (0.00)	1.22 (0.00)	38.65 (0.00)	0.75 (0.00)	38.78 (0.00)	1.01 (0.01)
Sputum test						
At week 9	1.11 (0.03)	1.05 (0.04)	6.81 (0.17)	6.12 (0.25)	3.64 (0.13)	3.27 (0.17)
At month 6	1.34 (0.01)	1.30 (0.02)	6.37 (0.31)	4.69 (0.18)	3.56 (0.16)	2.83 (0.12)
TB clinic	1.67 (0.05)	2.01 (0.09)	0.03 (0.01)	0.01 (0.01)	0.95 (0.04)	1.16 (0.08)
Anti-TB medicine	45.62 (0.26)	44.49 (0.62)	49.98 (0.78)	48.69 (1.38)	47.58 (0.38)	46.47 (0.70)
Doctor & hospital stay	5.58 (2.56)	5.68 (1.32)	45.45 (15.03)	61.26 (12.98)	29.23 (10.45)	33.32 (7.24)
Total	94.20 (2.54)	55.75 (1.51)	147.29 (14.91)	121.53 (13.11)	123.75 (10.42)	88.06 (7.30)
EQ-5D-5L utility, Mean (SE)						
Baseline	0.856 (0.009)	0.793 (0.016)	0.691 (0.017)	0.829 (0.014)	0.783 (0.010)	0.809 (0.011)
Month 6	0.944 (0.009)	0.901 (0.016)	0.848 (0.016)	0.842 (0.024)	0.901 (0.009)	0.875 (0.014)
QALYs	0.454 (0.004)	0.425 (0.007)	0.385 (0.007)	0.401 (0.011)	0.423 (0.004)	0.415 (0.006)
Adjusted incremental estimates, Mean (95% CI)						
Incremental costs^a^, INT$	38.35 (33.63–39.65)	–	28.68 (−12.79 to 65.55)	–	36.17 (3.65–65.81)	–
Incremental QALYs^b^	0.009 (−0.011 to 0.013)	–	0.028 (0.007–0.050)	–	0.017 (0.003–0.030)	–
ICER	INT$4261.11 per QALY gain (Uncertainty see Fig. 1a)	–	INT$1024.29 per QALY gain (Uncertainty see Fig. 1b)	–	INT$2127.64 per QALY gain (Uncertainty see Fig. 1c)	–

a For Bangladesh: adjusted for age with site as random intercepts (only one female); For Pakistan: adjusted for age, and sex with site as random intercepts; For all participants: adjusted for age, sex and country with site as random intercepts.

b Adjusted for age, EQ-5D-5L utility at baseline with site as random intercepts.

The intervention costed INT$138.86 (95% CI 99.18–249.87) more to achieve one additional abstainer (Table 4). Participants in the mTB-Tobacco group spent INT$40.74 (95% CI −114.37 to 35.13) less on care than those in the usual care group but lost INT$66.71 (95% CI −22.98 to 164.17) more through income. However, neither of them was significant.

**Table 4:** Secondary analyses: Incremental cost-effectiveness analysis for smoking cessation, and incremental analysis of participants’ spendings on care and lost income.

Costs, OOPs on care and lost income, INT$	Bangladesh		Pakistan		All participants	
	mTB-Tobacco (n = 400)	Usual care (n = 200)	mTB-Tobacco (n = 320)	Usual care (n = 160)	mTB-Tobacco (n = 720)	Usual care (n = 360)
Incremental treatment costs per abstainer						
Biochemically verified continuous abstinence at 6 months, % (n/N)	48.2% (189/392)	16.8% (32/191)	36.6% (111/303)	16.2% (23/142)	43.2% (300/695)	16.5% (55/333)
Intervention/control costs per abstainer, mean (95% CI)	80.68 (61.88–116.67)	7.26 (5.94–9.30)	105.60 (78.56–151.63)	4.63 (2.54–18.13)	89.77 (72.38–117.19)	6.12 (4.27–8.96)
Incremental, mean (95% CI)	119.97 (81.03–235.76)	–	185.78 (99.19–744.71)	–	138.86 (99.18–249.87)	–
Participants' spendings on care						
Baseline, mean (SE)						
TB clinic	14.62 (3.50)	1.38 (0.54)	3.21 (1.78)	5.43 (2.90)	9.55 (2.11)	3.18 (1.33)
Doctor & hospital stay	41.50 (7.80)	50.58 (7.26)	73.72 (11.92)	109.49 (16.61)	55.82 (6.86)	76.76 (8.54)
Total	56.12 (8.54)	51.96 (7.36)	76.93 (12.01)	114.91 (17.19)	65.37 (7.15)	79.94 (8.81)
6 months, mean (SE)						
TB clinic	28.10 (1.12)	23.67 (1.71)	0.66 (0.38)	1.38 (1.36)	15.82 (0.80)	16.59 (1.69)
Doctor & hospital stay	25.44 (11.24)	23.26 (6.27)	40.70 (15.42)	182.59 (27.93)	40.62 (14.48)	80.59 (12.53)
Total	53.54 (11.30)	46.93 (6.79)	41.36 (15.44)	183.97 (27.94)	56.44 (14.47)	97.18 (12.52)
Adjusted incremental spendings^a^, mean (95% CI)	6.61 (−8.01 to 21.51)	–	−142.61 (−160.73 to −87.32)	–	−40.74 (−114.37 to 35.13)	–
Participants' lost income						
Baseline, mean (SE)	75.38 (10.54)	123.90 (18.04)	131.51 (29.42)	52.56 (10.14)	100.33 (14.35)	92.19 (11.13)
6 months, mean (SE)	94.44 (13.65)	66.05 (10.69)	148.97 (36.40)	87.07 (13.29)	118.21 (18.77)	75.00 (8.14)
Adjusted incremental lost income^b^, mean (95% CI)	36.70 (15.95–65.29)	–	100.44 (−13.06 to 242.26)	–	66.71 (−22.98 to 164.17)	–

a By country: adjusted with site as random intercepts; For all participants: adjusted for country with site as random intercepts.

b For Bangladesh: adjusted for occupation, and lost income at baseline with site as random intercepts (only one female); For Pakistan: adjusted for sex, occupation, and lost income at baseline with site as random intercepts; For all participants: adjusted for sex, occupation, country and lost income at baseline with site as random intercepts.

The complete case analysis included 661 participants in the mTB-Tobacco group and 312 participants in the usual care group. Out of 107 participants excluded from complete case analysis, 52 were dead. From a public/voluntary sector perspective, the mTB-Tobacco was more costly but with no effects. The probability of mTB-Tobacco being cost-effective remained very low across WTP thresholds for Bangladesh and Pakistan. Other results based on complete cases remained consistent with those based on imputed data. Deviation from MAR assumption made a slight impact on incremental costs but negligible on incremental QALYs, spendings on care, and lost income. This resulted in increase in estimated ICERs ranging from INT$2203.69 to INT$2456.01 in all participants. The full results of sensitivity analyses were shown in Supplementary Material 5.

Results of the post-hoc country-specific analyses showed that the mTB-Tobacco were more costly and more effective in both Bangladesh and Pakistan (Table 3). The ICER in Bangladesh was higher than that in all participants whilst in Pakistan it was lower. From a public/voluntary sector perspective, mTB-Tobacco demonstrated a low likelihood of cost-effectiveness in Bangladesh but in Pakistan, the probability reached 95% at the upper boundary of WTP threshold. From participants’ perspective, the mTB-Tobacco produced divergent impacts between countries (Table 4). In Bangladesh, participants in the mTB-Tobacco group spent slightly more on care and lost more income than those in the usual care group. In Pakistan, mTB-Tobacco was associated with substantial reductions in spendings on care and an increase in lost income.

## Discussion

The present study found the mTB-Tobacco intervention was more costly and more effective than usual care, both in terms of biochemically verified continuous abstinence at 6 months and QALYs over 6 months, among newly diagnosed patients with TB and who smoked tobacco daily. The intervention becomes likely cost-effective only when the WTP threshold approaches the upper boundary of selected thresholds.

There was a lack of evidence on cost-effectiveness of smoking cessation interventions among people with TB in LMICs. In Bangladesh and Pakistan, only one other study compared cytisine plus brief behavioural support and brief behavioural support alone.^26^ It reported cytisine plus brief behavioural support costed INT$58 more than brief behavioural support alone, with no effects in terms of QALYs over 6 months, concluding cytisine was not cost-effective. In contrast, the incremental costs in our study were smaller, even more so when inflating from 2018 prices, and we reported a significant gain of QALYs in the mTB-Tobacco group. This difference might be partly due to the significant difference in the number of recorded deaths between groups in our study. Null difference in QALYs between groups in the complete case analysis appears to support this observation. The biochemically verified continuous abstinence rates at 6 months of the citysine study were around 30% in both groups,^26^ lower than that in the mTB-Tobacco group but nearly doubled that in the usual care group in our study. This led to a different conclusion of cost-effectiveness in terms of smoking cessation.

Whilst the consensus is that automated text-messaging interventions are effective in smoking cessation and offers a potentially scalable solution at relatively low costs, the evidence on cost-effectiveness and sustainability is lacking for a successful implementation in LMICs.^7^ Our results align with the consensus whilst showed a high level of uncertainty in cost-effectiveness.

First, the mTB-Tobacco intervention might be cheaper than pharmacotherapies for smoking cessation in LMICs but was far more expensive than two sessions of brief behavioural support (INT$12 per participant^26^). Brief behavioural support incurred less than one-third of the costs of mTB-Tobacco yet yielded 70% of the abstainers achieved by the latter. This indicates that mobile-base interventions may fail to achieve cost-effectiveness in low-labour-cost contexts. However, face-to-face interventions rely on personnel capacity, which lacks the potential for rapid expansion. In countries where the resources and capacity are constrained, policymakers may face a trade-off between the efficiency of digital scale-ups and the economic sustainability of using local human capital.

Second, our findings suggest that among LMICs, the key drivers of cost-effectiveness can vary. Country-specific analyses underscored the divergent trends present within the pooled data. In Bangladesh, the incremental costs were almost exactly the cost of mTB-Tobacco whilst the difference in QALYs was marginal. In Pakistan, with a significant QALY gain by mTB-Tobacco, the costs of intervention were partially offset by the lower costs of doctor and hospital stay in the mTB-Tobacco group. However, it should be noted that the sample size was not estimated by country, and these findings should be interpreted with caution.

The strength of this study is reflected in 90% retention of participants at follow-up. Krishnan et al. identified 12 articles assessing text-message based intervention for smoking cessation in LMICs and concluded that the intervention could be effective but more RCTs with large sample sizes, longer follow-up, and biochemically verified cessation, are needed to establish efficacy.^27^

Sensitivity analyses demonstrated that exclusion of missing data reduced the difference between groups, thus erasing the probability of mTB-Tobacco being cost-effective. The exclusion of deaths occurring during follow-up may significantly contribute to this impact. Missing due to ill health increased the difference between groups but this effect was negligible on incremental QALYs. As a result, the ICER increased but the extent to which was moderate.

Several limitations exist in the present study. Although we accounted for cluster-level variance via random intercepts, a limitation of the analysis is that the correlation between costs and QALYs was not explicitly modelled. This may affect the results if the actual level of correlation was high. Our results are also limited by the short follow-up period. Averted treatment costs and QALYs gained beyond 6 months were not included. As the risk of recurrence or relapse of TB and incidence of smoking-related diseases among those who continued to smoke is higher than those who quit,^4^^,^^28^ in the long-term the mTB-Tobacco intervention could become more cost-effective. For instance, one study conducted in the UK on smoking cessation reported 6-month within trial ICER at £7750 per QALY gained and model projected lifetime ICER at £1131 per QALY gained when the difference in 6-month biochemically verified continuous abstinence was 3.1%.^29^

In our study setting, over one third of the intervention costs were accounted for by support staff who monitored the messaging system, and the short text messages were costed using a fixed price per message, both of which were charged according to the rates agreed by the third-party contractor. Outside of the study settings, it may be possible to negotiate a lower rate for text messages or assign the monitoring task to an existing IT team, especially if the mTB-Tobacco intervention were to be commissioned and implemented by National TB Programme. On the other hand, our findings did not include administrative costs which would be required in implementation.

Due to the lack of validated valuation tariff for the two countries, we used the Indian tariff to covert EQ-5D-5L domain profile to utility values. This added a layer of uncertainty to the conclusion. However, in a previous study conducted in these countries, sensitivity analysis using valuation tariffs from several countries suggested that though the absolute values of utilities indeed varied with the tariff used, the difference in relative values (i.e. incremental QALYs) was not sufficiently large to change the conclusion.^26^ The thresholds used to determine cost-effectiveness were estimated based on an article published nearly ten years ago.^25^ Simple inflation might not be sufficient to fully account for relevant changes in recent years. Whilst this provided some references, the individual countries may have their own priorities set in this regard.

Our study was conducted in two LMICs where people are likely to pay privately for healthcare services at the point of use,^30^ which would influence their behaviours in seeking care and complicate the choice of the perspective to assess cost-effectiveness of smoking cessation interventions. Those diagnosed with TB and who smoke tobacco would benefit not only in health but also in reduced household healthcare expenditure. However, they would be unlikely to fund a smoking cessation programme such as mTB-Tobacco, which represents the standard perspective adopted in conventional economic evaluations. Whilst the societal perspective would be most comprehensive, it remains a theoretical entity. In practice, fundings for specific programmes is generally drawn from the allocated budgets of individual institutions. They typically focus on institution-specific returns, often disregarding wider benefits that do not directly affect them. The participants were predominantly male, especially in Bangladesh, and belonging to middle-age. This limits generalisability to the female population.

In conclusion, the mTB-Tobacco intervention was more effective but also more costly among pulmonary TB patients in Bangladesh and Pakistan. It is cost-effective only if decision makers are willing to pay over INT$2100 for one additional QALY gained. Future research in LMICs should further investigate relative effectiveness of low-intensity face-to-face smoking cessation interventions compared to mTB-Tobacco. Localised implementation and evaluation of such implementation is essential. It is also crucial to capture recurrence or relapse of TB and incidence of smoking related diseases beyond 6 months to fully assess costs and benefits of abstinence among people with TB who smoke tobacco.

## Contributors

JL and SP had directly accessed and verified the underlying data that used in this manuscript. FR, MD, SA, SP, RH, JN, and KS conceptualised and designed the study. MZ, FR, MD, SHR, AC, AKL, MB, JL, AK, RH, and KS performed data curation. JL and SP designed and carried out the analysis. AKL, SP, JN, and KS acquired the funding for this study. MZ, FR, AKL, MB, AK, and RH oversaw and managed resources for the study. SP, AKL, AK, RH, and KS supervised the overall procedures of the study. JL and SP drafted the initial manuscript of this study. JL, SP, AC, AKL, MB, AK, MZ, and KS participated in review and editing of the final manuscript. All authors have access to all the data reported in the study.All authors have read and approved the final version of the manuscript.

## Data sharing statement

The trial registration number is ISRCTN 861971818. Registry webpage is https://www.isrctn.com/ISRCTN86971818. De-identified participant datasets generated and/or analysed during the current study will be stored in secure, password-protected data repositories (DataVault or DataShare) hosted by the University of Edinburgh for a minimum of 10 years following the end of the study. DataShare is an open-access repository for anonymised data, which means that all non-identifiable data is freely available. DataVault is a secure repository for sensitive information which can only be accessed by approved researchers who have undergone a rigorous application and review process.

## Declaration of interests

The University of Edinburgh is the contracting organisation of the RESPIRE-II. JN was employed at the University of Edinburgh until 1 Aug 2024 on this project. AKL is employed by the University of Edinburgh on this project. ARK Foundation is a partner of the RESPIRE-II. RH is employed at the ARK Foundation, Bangladesh on this project. The Initiative is a partner of the RESPIRE-II. AK is employed at The Initiative, Pakistan on this project. University of York is a partner of the RESPIRE. JL is employed by University of York on this project. KS reported receiving consulting fees from Perrigo outside the submitted work. All other authors declare no conflict of interest.

## Appendix A Supplementary data

Supplementary Figures and Tables

Bmjopen
