# Supplementary material for: Cost-effectiveness of a mTB-Tobacco intervention for smoking cessation in people with tuberculosis: an economic evaluation of a cluster randomised controlled trial
Source: Lancet Reg Health Southeast Asia. 2026 May 5;49:100776. doi: 10.1016/j.lansea.2026.100776 (PMC13158790; doi:10.1016/j.lansea.2026.100776)
Supplement: Supplementary Figures and Tables [file mmc1.pdf]

Supplementary materials of Cost-effectiveness of a mTB-Tobacco intervention for smoking cessation in people with tuberculosis: Economic evaluation of a cluster randomised controlled trial

Table of Contents

Supplementary material 1 Details of unit costs with additional references 3

Additional references 3

Supplementary material 2 Missing data pattern 5

Supplementary material 3 Detailed summary of data collected at baseline and 6 months 7

TB treatment and healthcare costs 7

Participants' spending for TB treatment and healthcare services 8

Participants' days off work and lost income 9

EQ-5D-5L 10

Supplementary material 4 Supplementary results of the primary analysis 12

Supplementary material 5 Sensitivity analyses: complete case analysis and MNAR examinations 14

Supplementary table 1 Unit costs of TB treatment and wider healthcare in Bangladesh and Pakistan (INT$ 2024) 3

Supplementary table 2 Average income per hour by sex and occupational categories in Bangladesh and Pakistan (INT$ 2024) 3

Supplementary table 9 Missing data table by groups and total 5

Supplementary table 10 Association of missingness with baseline covariates examined by univariate logistic regression 6

Supplementary table 3 Breakdown of costs of TB treatment, by groups 7

Supplementary table 4 Mean number of uses and mean costs of healthcare services at baseline and 6 months, by groups 7

Supplementary table 5 Mean spendings for TB treatment by participants at baseline and 6 months, by groups ... 8

Supplementary table 6 Mean spendings for healthcare services by participants at baseline and 6 months, by groups 8

Supplementary table 7 Mean number of days off work and mean lost income at baseline and 6 months, by groups 9

Supplementary table 8 Mean EQ-5D-5L utility value and VAS score at baseline and 6 months, by groups 10

Supplementary table 11 Association of missingness with baseline covariates examined by χ^2 test 11

Supplementary table 12 Association of missingness with values of the same measure at other data collection points, examined by univariate logistic regression 11

Supplementary figure 1 Sensitivity analysis: Cost-effectiveness planes (CEPs) showing the distribution of bootstrapped incremental costs and QALYs of mTB-Tobacco being cost-effective compared to usual care based on the primary analysis (n=1,080) 13

Supplementary table 13 Sensitivity analysis: Complete case analyses of incremental cost-utility analysis, incremental cost-effectiveness analysis for smoking cessation, and incremental analysis of participants' spendings and lost income 14

Supplementary figure 2 Sensitivity analysis: Cost-effectiveness acceptability curve (CEAC) showing the probability of mTB-Tobacco being cost-effective compared to usual care based on the complete case analysis (n=973) 15

Supplementary figure 3 Sensitivity analysis: Cost-effectiveness planes (CEPs) showing the distribution of bootstrapped incremental costs and QALYs of mTB-Tobacco being cost-effective compared to usual care based on the complete case analysis (n=973) 16
Supplementary table 14 Sensitivity analysis: Incremental estimates of costs, spendings on care, lost income and QALYs under MNAR scenarios 17

Supplementary material 1 Details of unit costs with additional references

Costs of anti-TB medicine were estimated by applying the average costs of anti-TB medicine per patient for drug sensitive TB to the level of completion based on information from routine TB treatment card. Global TB report in 2024 reported the average costs spent per patient in 2023,[1] which was inflated to INT$46·33 in Bangladesh and INT$56·62 in Pakistan.[2] The costs per sputum test were INT$1·36 in Bangladesh and INT$5·78 in Pakistan, derived from published literature,[3,4] converted and inflated to INT$ 2024.[2,5,6]

The unit costs per TB clinic visit were estimated at INT$0·74 in Bangladesh and INT$0·96 in Pakistan using provider costs per patient,[4,7] divided by average number of visits per patient[1] and inflated to 2024.[2,5,6] The unit costs per doctor visit were INT$12·60 in Bangladesh and INT$32·57 in Pakistan and the unit costs per day of hospital stay were INT$49·76 in Bangladesh and INT$179·07, both of which were derived from published literature [8-11], inflated to 2024 and converted to INT$.[2,5,6] Supplementary table 1 summarises the unit costs.

Supplementary table 1 Unit costs of TB treatment and wider healthcare in Bangladesh and Pakistan (INT$ 2024)

Unit costs (INT$)	Bangladesh	Pakistan
**TB-Treatment**		
**Anti-TB medicine/patient**	46·33	56·62
**Sputum test/test**	1·36/	5·78
**TB clinic/visit**	0·74	0·96
**Wider healthcare**		
**Doctor/visit**	12·60	32·57
**Hospital stay/day**	49·76	179·07

Participants' lost income was estimated by applying average income to the number of days off work. The average income by sex and occupational categories was extracted from Labour Force Survey in both countries [12,13], then inflated and converted to INT$ 2024 (Supplementary table 2).[2,6] If participants reported not in employment at baseline but had a job at 6 months, overall average of all categories was used to estimate their loss of income. If they did not have a job at the time or did not take any days off work, they were considered not having lost any income.

Supplementary table 2 Average income per hour by sex and occupational categories in Bangladesh and Pakistan (INT$ 2024)

Income INT$/hour by occupational categories	Bangladesh			Pakistan		
	**Male**	**Female**	**Total**	**Male**	**Female**	**Total**
Managers	6·51	7·22	6·46	9·06	10·16	9·06
Professionals	4·71	4·93	4·78	7·13	5·89	6·60
Technicians and Associate Professionals	3·58	3·94	3·62	4·57	4·23	4·51
Clerical support workers	3·95	3·81	3·94	4·81	5·07	4·82
Services and Sale workers	2·24	2·10	2·24	2·39	2·57	2·40
Skilled Agricultural, Forestry and Fisheries	2·07	2·67	2·41	3·63	3·15	4·25
Craft and related trade workers	2·36	2·30	2·32	2·79	2·20	2·80
Plant and Machine Operators, and Assembler	2·32	1·71	2·27	2·58	3·33	2·58
Elementary Occupation	2·04	1·71	1·98	2·36	1·88	2·35
Other Occupations	4·83	4·06	4·84	-	-	-
**Total**	2·69	3·09	2·75	3·23	4·16	3·44

Additional references

1. World Health Organization (WHO). Global Tuberculosis Report 2024. Geneva, 2024. https://www.who.int/teams/global-programme-on-tuberculosis-and-lung-health/tb-reports/global-tuberculosis-report-2024 (accessed 20/02/2025).

2. International Monetary Fund. Prices, Production and Laor selected indicators. 2024. https://data.imf.org (accessed 20 Feb 2025).

3. Hasan MZ, Ahmed S, Islam Z, et al. Costs of services and funding gap of the Bangladesh National Tuberculosis Control Programme 2016-2022: An ingredient based approach. *PloS one* 2023; **18**(6): e0286560. 10.1371/journal.pone.0286560.

4. Hussain H, Mori AT, Khan AJ, et al. The cost-effectiveness of incentive-based active case finding for tuberculosis (TB) control in the private sector Karachi, Pakistan. *BMC health services research* 2019; **19**(1): 690. 10.1186/s12913-019-4444-z.
5. International Monetary Fund. Exchange Rates selected indicators. 2023. https://data.imf.org (accessed 23/06/2023).
6. International Monetary Fund (IMF). Implied PPP conversion rate - National currency per international dollar. 2025. https://www.imf.org/external/datamapper/PPPEX@WEO/BGD/PAK (accessed 20 Feb 2025).
7. Haider MR. Economic Burden of Tuberculosis among Bangladeshi Population and Economic Evaluation of the Current Approaches of Tuberculosis Control in Bangladesh [Doctor dissertation]. Columbia: University of South Carolina; 2017.
8. Zeng W, Halasa YA, Cros M, Akhter H, Nandakumar AK, Shepard DS. Costing essential services package provided by a non-governmental organization network in Bangladesh. *Health Policy Plan* 2017; **32**(10): 1375–85. 10.1093/heapol/czx105.
9. Alvi MH, Shiri T, Iqbal N, et al. Cost-Effectiveness of a Culturally Adapted Manual-Assisted Brief Psychological Intervention for Self-Harm in Pakistan: A Secondary Analysis of the Culturally Adapted Manual-Assisted Brief Psychological Randomized Controlled Trial. *Value Health Reg Issues* 2022; **27**: 65–71. 10.1016/j.vhri.2021.08.005.
10. Mejia N, Pallas SW, Saha S, et al. Typhoid and Paratyphoid Cost of Illness in Bangladesh: Patient and Health Facility Costs From the Surveillance for Enteric Fever in Asia Project II. *Clin Infect Dis* 2020; **71**(Suppl 3): S293–S305. 10.1093/cid/ciaa1334.
11. Mejia N, Qamar F, Yousafzai MT, et al. Typhoid and Paratyphoid Cost of Illness in Pakistan: Patient and Health Facility Costs From the Surveillance for Enteric Fever in Asia Project II. *Clin Infect Dis* 2020; **71**(Suppl 3): S319–S35. 10.1093/cid/ciaa1336.
12. Bangladesh Bureau of Statistics. Labour Force Survey 2022 Bangladesh, 2023. https://webapps.ilo.org/surveyLib/index.php/catalog/8538/study-description (accessed 24 Feb 2025).
13. Pakistan Bureau of Statistics. Pakistan Labour Force Survey 2020-21, 2022. https://www.pbs.gov.pk/content/labour-force-statistics (accessed 24 Feb 2025).

Supplementary material 2 Missing data pattern

Supplementary table 3 Missing data table by groups and total

	Bangladesh						Pakistan						All participants					
	mTB-Tobacco (n=400)		Usual care (n=200)		Total (n=600)		mTB-Tobacco (n=320)		Usual care (n=160)		Total (n=480)		mTB-Tobacco (n=720)		Usual care (n=360)		Total (n=1,080)	
**Missing**	**n**	**%**	**n**	**%**	**n**	**%**	**n**	**%**	**n**	**%**	**n**	**%**	**n**	**%**	**n**	**%**	**n**	**%**
Age	0	0%	0	0%	0	0%	0	0%	0	0%	0	0%	0	0%	0	0%	0	0%
Sex	0	0%	0	0%	0	0%	0	0%	0	0%	0	0%	0	0%	0	0%	0	0%
Country	0	0%	0	0%	0	0%	0	0%	0	0%	0	0%	0	0%	0	0%	0	0%
Site	0	0%	0	0%	0	0%	0	0%	0	0%	0	0%	0	0%	0	0%	0	0%
**Baseline**																		
Costs of sputum tests	0	0%	0	0%	0	0%	0	0%	0	0%	0	0%	0	0%	0	0%	0	0%
Costs of TB clinic	0	0%	0	0%	0	0%	0	0%	0	0%	0	0%	0	0%	0	0%	0	0%
Costs of doctors & hospital stays	1	0%	0	0%	1	0%	0	0%	0	0%	0	0%	1	0%	0	0%	1	0%
Expenses on TB clinic visits	0	0%	0	0%	0	0%	0	0%	0	0%	0	0%	0	0%	0	0%	0	0%
Expenses on doctor & hospital stays	1	0%	0	0%	1	0%	1	0%	0	0%	1	0%	2	0%	0	0%	2	0%
Expenses on tobacco	0	0%	0	0%	0	0%	0	0%	0	0%	0	0%	0	0%	0	0%	0	0%
Lost income due to sick leave	0	0%	0	0%	0	0%	0	0%	0	0%	0	0%	0	0%	0	0%	0	0%
EQ-5D-5L utility	0	0%	0	0%	0	0%	0	0%	0	0%	0	0%	0	0%	0	0%	0	0%
Costs of mTB-Tobacco/Usual care	0	0%	0	0%	0	0%	0	0%	0	0%	0	0%	0	0%	0	0%	0	0%
**Week 9**																		
Costs of sputum tests	0	0%	1	0%	1	0%	10	3%	9	6%	19	4%	10	1%	10	3%	20	2%
**Month 6**																		
Costs of sputum tests	11	3%	6	3%	17	3%	17	5%	9	6%	26	5%	28	4%	15	4%	43	4%
Costs of TB clinic	19	5%	15	8%	34	6%	34	11%	27	17%	61	13%	53	7%	42	12%	95	9%
Costs of anti-TB medicines	11	3%	6	3%	17	3%	17	5%	9	6%	26	5%	28	4%	15	4%	43	4%
Costs of doctors & hospital stays	19	5%	15	8%	34	6%	34	11%	27	17%	61	13%	53	7%	42	12%	95	9%
Expenses on TB clinic visits	19	5%	15	8%	34	6%	34	11%	27	17%	61	13%	53	7%	42	12%	95	9%
Expenses on doctor & hospital stays	19	5%	15	8%	34	6%	35	11%	28	17%	63	13%	54	8%	43	12%	97	9%
Expenses on tobacco	20	5%	15	8%	35	6%	74	23%	31	19%	105	22%	94	13%	46	13%	140	13%
Lost income due to sick leave	19	5%	15	8%	34	6%	34	11%	28	18%	62	13%	53	7%	43	12%	96	9%
EQ-5D-5L utility	11	3%	6	3%	17	3%	17	5%	9	6%	26	5%	28	4%	15	4%	43	4%
Treatment outcome	0	0%	0	0%	0	0%	0	0%	0	0%	0	0%	0	0%	0	0%	0	0%

Supplementary table 4 Association of missingness with baseline covariates examined by univariate logistic regression

**Univariate logistic regression OR (Z, p)**							
**Missing on:**	**Allocation**	**Country**	**Age**	**Sex**	**Number of pre-existing illness**	**Costs of doctor & hospital stays**	**EQ-5D-utility**
**Week 9**							
Costs of sputum tests	2·03 (Z=1·57, p=0·118)	**24·69 (Z=3·12, p=0·002)**	1·01 (Z=0·83, p=0·404)	1·50 (Z=0·39, p=0·698)	0·85 (Z=-0·24, p=0·807)	1·00 (Z=0·00, p=0·997)	2·59 (Z=0·79, p=0·431)
**Month 6**							
Costs of sputum tests	1·07 (Z=0·22, p=0·826)	**1·96 (Z=2·12, p=0·034)**	1·01 (Z=0·50, p=0·614)	1·40 (Z=0·45, p=0·654)	1·16 (Z=0·37, p=0·709)	0·99 (Z=-1·13, p=0·258)	1·47 (Z=0·50, p=0·617)
Costs of TB clinic	**1·66 (Z=2·34, p=0·020)**	**2·42 (Z=3·96, p=0·000)**	**1·03 (Z=3·56, p=0·000)**	0·91 (Z=-0·15, p=0·881)	1·24 (Z=0·79, p=0·429)	1·00 (Z=0·02, p=0·983)	**0·33 (Z=-2·45, p=0·014)**
Costs of anti-TB medicines	1·07 (Z=0·22, p=0·826)	**1·96 (Z=2·12, p=0·034)**	1·01 (Z=0·50, p=0·614)	1·40 (Z=0·45, p=0·654)	1·16 (Z=0·37, p=0·709)	0·99 (Z=-1·13, p=0·258)	1·47 (Z=0·50, p=0·617)
Costs of doctors & hospital stays	**1·66 (Z=2·34, p=0·020)**	**2·42 (Z=3·96, p=0·000)**	**1·03 (Z=3·56, p=0·000)**	0·91 (Z=-0·15, p=0·881)	1·24 (Z=0·79, p=0·429)	1·00 (Z=0·02, p=0·983)	**0·33 (Z=-2·45, p=0·014)**
Expenses on TB clinic visits	**1·66 (Z=2·34, p=0·020)**	**2·42 (Z=3·96, p=0·000)**	**1·03 (Z=3·56, p=0·000)**	0·91 (Z=-0·15, p=0·881)	1·24 (Z=0·79, p=0·429)	1·00 (Z=0·02, p=0·983)	**0·33 (Z=-2·45, p=0·014)**
Expenses on doctor & hospital stays	**1·67 (Z=2·39, p=0·017)**	**2·52 (Z=4·15, p=0·000)**	**1·03 (Z=3·63, p=0·000)**	1·24 (Z=0·40, p=0·693)	1·30 (Z=1·01, p=0·314)	1·00 (Z=0·12, p=0·904)	**0·33 (Z=-2·52, p=0·012)**
Expenses on tobacco	0·98 (Z=-0·13, p=0·898)	**4·52 (Z=7·31, p=0·000)**	**1·02 (Z=3·91, p=0·000)**	1·05 (Z=0·10, p=0·919)	1·21 (Z=0·82, p=0·410)	1·00 (Z=-0·29, p=0·768)	**0·34 (Z=-2·82, p=0·005)**
Lost income due to sick leave	**1·71 (Z=2·47, p=0·013)**	**2·47 (Z=4·05, p=0·000)**	**1·02 (Z=3·45, p=0·001)**	0·90 (Z=-0·17, p=0·865)	1·23 (Z=0·75, p=0·452)	1·00 (Z=0·00, p=0·997)	**0·34 (Z=-2·40, p=0·017)**
EQ-5D-5L utility	1·07 (Z=0·22, p=0·826)	**1·96 (Z=2·12, p=0·034)**	1·01 (Z=0·50, p=0·614)	1·40 (Z=0·45, p=0·654)	1·16 (Z=0·37, p=0·709)	0·99 (Z=-1·13, p=0·258)	1·47 (Z=0·50, p=0·617)

OR (Z, p) in bold font denote a significant difference.

The association of missingness of each measure with group allocation and baseline covariates, and with observed values of the same measure at other data collection points was examined using statistical tests (univariable logistic regression for continuous and binary variables, χ^2 tests for discrete variables) with 0·05 as significant level. An imputation model was developed, including all the measures necessary for the analysis or associated with missingness identified by the statistical tests, along with days in the study and death status (survived to 6 months, died before 9 weeks, and died between 9 weeks and 6 months).

Supplementary material 3 Detailed summary of data collected at baseline and 6 months

TB treatment and healthcare costs

Supplementary table 5 Breakdown of costs of TB treatment, by groups

	mTB-Tobacco			Usual care		
	**n**	**Mean number of uses or proportion (SD)**	**Mean costs (SD), INT$**	**n**	**Mean number of uses or proportion (SD)**	**Mean costs (SD), INT$**
**Bangladesh**		**n=400**			**n=200**	
**Anti-TB medicines***						
Completion	389	98% (11%)	45·60 (5·19)	194	96% (19%)	44·47 (8·68)
**Sputum tests***						
Baseline	400	1·03 (0·44)	1·39 (0·59)	200	1·09 (0·43)	1·48 (0·58)
Week 9	400	0·81 (0·42)	1·11 (0·57)	199	0·77 (0·47)	1·05 (0·63)
Month 6	389	0·98 (0·15)	1·34 (0·21)	194	0·95 (0·21)	1·30 (0·29)
**Visits to TB clinic**						
Baseline	400	0·40 (0·94)	0·30 (0·70)	200	0·16 (0·51)	0·11 (0·38)
Month 6	381	2·25 (1·19)	1·67 (0·88)	185	2·71 (1·36)	2·00 (1·01)
**Pakistan**		**n=320**			**n=160**	
**Anti-TB medicines***						
Completion	303	88% (24%)	49·93 (13·82)	151	86% (31%)	48·50 (17·54)
**Sputum tests***						
Baseline	320	1·02 (0·17)	5·91 (0·96)	160	1·00 (0·00)	5·78 (0·00)
Week 9	310	1·18 (0·53)	6·81 (3·04)	151	1·06 (0·53)	6·12 (3·08)
Month 6	303	1·10 (0·92)	6·37 (5·34)	151	0·81 (0·39)	4·71 (2·25)
**Visits to TB clinic**						
Baseline	320	0·08 (0·50)	0·07 (0·48)	160	0·18 (1·08)	0·17 (1·04)
Month 6	286	0·03 (0·22)	0·03 (0·21)	133	0·01 (0·09)	0·01 (0·08)
**All participants**		**n=720**			**n=360**	
**Anti-TB medicines***						
Completion	692	94% (19%)	47·50 (10·16)	345	92% (25%)	46·23 (13·43)
**Sputum tests***						
Baseline	720	1·02 (0·34)	3·40 (2·38)	360	1·05 (0·32)	3·39 (2·18)
Week 9	710	0·97 (0·50)	3·59 (3·49)	350	0·90 (0·51)	3·24 (3·26)
Month 6	692	1·04 (0·62)	3·54 (4·33)	345	0·89 (0·31)	2·79 (2·27)
**Visits to TB clinic**						
Baseline	720	0·26 (0·80)	0·20 (0·62)	360	0·16 (0·82)	0·14 (0·75)
Month 6	667	1·30 (1·43)	0·96 (1·06)	318	1·58 (1·69)	1·17 (1·25)

*Information from TB cards were available for the 25 participants (8 in Bangladesh, 17 in Pakistan) who died after week 9. Costs of medicines and sputum tests for the 27 participants (9 in Bangladesh, 18 in Pakistan) died before week 9 were assumed to be null.

Supplementary table 6 Mean number of uses and mean costs of healthcare services at baseline and 6 months, by groups

	mTB-Tobacco			Usual care		
**Healthcare costs**	**n**	**Mean number of uses (SD)**	**Mean costs (SD), INT$**	**n**	**Mean number of uses (SD)**	**Mean costs (SD), INT$**
**Bangladesh**		**n=400**			**n=200**	
**Baseline**						
Public doctor	400	0·25 (0·68)	3·15 (8·63)	200	0·60 (1·04)	7·56 (13·07)
Public hospital stay (days)	399	0·09 (0·65)	4·61 (32·29)	200	0·09 (0·56)	4·23 (27·67)
Total	399	-	7·77 (33·41)	200	-	11·79 (33·72)
**Month 6**						
Public doctor	381	0·18 (0·54)	2·25 (6·84)	185	0·38 (0·79)	4·77 (9·98)
Public hospital stay (days)	381	0·05 (0·67)	2·35 (33·20)	185	0·02 (0·29)	1·08 (14·63)
Total	381	-	4·60 (33·74)	185	-	5·84 (18·92)
**Pakistan**		**n=320**			**n=160**	
**Baseline**						
Public doctor	320	0·74 (3·12)	24·02 (101·73)	160	1·11 (1·80)	36·23 (58·79)
Public hospital stay (days)	320	0·12 (1·29)	21·82 (230·43)	160	0·09 (0·78)	15·67 (139·70)
Total	320	-	45·84 (253·14)	160	-	51·90 (158·99)
**Month 6**						
Public doctor	286	0·24 (1·03)	7·97 (33·46)	133	1·83 (3·20)	59·74 (104·25)
Public hospital stay (days)	286	0·15 (1·01)	26·92 (180·18)	133	0·07 (0·78)	12·12 (139·75)
Total	286	-	34·89 (182·86)	133	-	71·87 (170·11)
**All participants**		**n=720**			**n=360**	

**Baseline**						
Public doctor	720	0·47 (2·16)	12·43 (68·85)	360	0·83 (1·45)	20·30 (42·76)
Public hospital stay (days)	719	0·11 (0·99)	12·27 (155·70)	360	0·09 (0·66)	9·31 (95·40)
Total	719	-	24·72 (171·60)	360	-	29·62 (110·56)
**Month 6**						
Public doctor	667	0·21 (0·79)	4·70 (22·67)	318	0·99 (2·27)	27·76 (72·95)
Public hospital stay (days)	667	0·09 (0·83)	12·89 (121·12)	318	0·04 (0·55)	5·69 (91·03)
Total	667	-	17·59 (123·22)	318	-	33·46 (115·42)

Participants' spending for TB treatment and healthcare services

Supplementary table 7 Mean spendings for TB treatment by participants at baseline and 6 months, by groups

	mTB-Tobacco		Usual care	
	**n**	**Mean (SD), INT$**	**n**	**Mean (SD), INT$**
**Bangladesh**	**n=400**		**n=200**	
**Baseline**				
Fees for visits TB clinic	400	9·67 (51·31)	200	0·53 (4·17)
Travel expense for visits to TB clinic				
Participants	400	2·71 (11·97)	200	0·52 (2·29)
Companion to participant	400	2·23 (11·72)	200	0·33 (1·91)
Total spending	400	14·62 (69·93)	200	1·38 (7·61)
**Month 6**				
Fees for visits TB clinic	381	15·24 (15·55)	185	11·12 (19·70)
Travel expense for visits to TB clinic				
Participants	381	8·97 (6·67)	185	10·24 (6·90)
Companion to participant	381	4·01 (6·23)	185	2·45 (4·00)
Total spending	381	28·21 (20·81)	185	23·81 (23·00)
**Pakistan**	**n=320**		**n=160**	
**Baseline**				
Fees for visits TB clinic	320	2·33 (30·23)	160	2·17 (14·59)
Travel expense for visits to TB clinic				
Participants	320	0·54 (3·88)	160	2·23 (15·69)
Companion to participant	320	0·33 (2·62)	160	1·03 (7·00)
Total spending	320	3·21 (31·79)	160	5·43 (36·72)
**Month 6**				
Fees for visits TB clinic	286	0·06 (0·67)	133	1·12 (12·96)
Travel expense for visits to TB clinic				
Participants	286	0·41 (3·59)	133	0·11 (1·30)
Companion to participant	286	0·17 (2·66)	133	0·22 (2·59)
Total spending	286	0·64 (6·04)	133	1·46 (16·84)
**All participants**	**n=720**		**n=360**	
**Baseline**				
Fees for visits TB clinic	720	6·42 (43·36)	360	1·26 (10·23)
Travel expense for visits to TB clinic				
Participants	720	1·74 (9·35)	360	1·28 (10·61)
Companion to participant	720	1·39 (8·96)	360	0·64 (4·88)
Total spending	720	9·55 (56·52)	360	3·18 (25·16)
**Month 6**				
Fees for visits TB clinic	667	8·73 (13·95)	318	6·94 (17·88)
Travel expense for visits to TB clinic				
Participants	667	5·30 (6·99)	318	6·01 (7·31)
Companion to participant	667	2·36 (5·37)	318	1·52 (3·64)
Total spending	667	16·39 (21·20)	318	14·47 (23·39)

Supplementary table 8 Mean spendings for healthcare services by participants at baseline and 6 months, by groups

	mTB-Tobacco			Usual care		
**Healthcare expenses**	**n**	**Mean number of uses (SD)**	**Mean spendings (SD), INT$**	**n**	**Mean number of uses (SD)**	**Mean spendings (SD), INT$**
**Bangladesh**	**n=400**			**n=200**		
**Baseline**						
Doctor	400	-	33·15 (137·04)	200	-	47·69 (97·26)
Public	400	0·25 (0·68)	8·49 (38·13)	200	0·60 (1·04)	10·36 (36·60)

Private	400	0·20 (0·79)	18·34 (112·08)	200	0·35 (0·76)	29·14 (75·31)
Travel patient	400	-	3·58 (15·42)	200	-	5·51 (10·16)
Travel companion	400	-	2·74 (11·59)	200	-	2·67 (6·39)
Hospital stay (days)	399	-	8·26 (63·26)	200	-	2·89 (19·46)
Public	399	0·09 (0·65)	7·84 (62·74)	200	0·09 (0·56)	2·38 (16·98)
Private	399	0·01 (0·10)	0·42 (8·46)	200	0·01 (0·14)	0·51 (7·17)
Total	399	-	41·49 (156·25)	200	-	50·58 (102·61)
**Month 6**						
Doctor	381	-	19·25 (102·22)	185	-	16·38 (46·91)
Public	381	0·18 (0·54)	3·85 (12·62)	185	0·38 (0·79)	5·78 (13·26)
Private	381	0·11 (0·55)	11·79 (90·01)	185	0·11 (0·50)	7·62 (34·78)
Travel patient	381	-	2·14 (9·24)	185	-	2·32 (5·12)
Travel companion	381	-	1·46 (5·16)	185	-	0·65 (2·86)
Hospital stay (days)	381	-	3·73 (61·30)	185	-	2·63 (30·39)
Public	381	0·05 (0·67)	0·62 (9·32)	185	0·02 (0·29)	0·44 (5·97)
Private	381	0·00 (0·05)	3·11 (60·62)	185	0·32 (4·41)	2·19 (29·83)
Total	381	-	22·97 (150·30)	185	-	19·01 (57·16)
**Pakistan**		**n=320**			**n=160**	
**Baseline**						
Doctor	319	-	72·89 (212·77)	160	-	108·37 (210·32)
Public	320	0·74 (3·12)	2·69 (19·80)	160	1·11 (1·80)	8·90 (39·41)
Private	319	2·40 (5·40)	44·79 (147·84)	160	1·20 (2·28)	39·01 (92·08)
Travel patient	320	-	16·82 (50·81)	160	-	41·32 (80·77)
Travel companion	320	-	9·54 (38·85)	160	-	19·13 (37·64)
Hospital stay (days)	320	-	0·79 (8·18)	160	-	1·12 (10·13)
Public	320	0·12 (1·29)	0·30 (4·23)	160	0·09 (0·78)	1·12 (10·13)
Private	320	0·06 (0·62)	0·49 (6·89)	160	0·00 (0·00)	0·00 (0·00)
Total	319	-	73·68 (213·48)	160	-	109·49 (210·15)
**Month 6**						
Doctor	285	-	32·15 (164·73)	132	-	153·80 (264·21)
Public	286	0·24 (1·03)	1·88 (13·18)	133	1·83 (3·20)	4·50 (7·66)
Private	285	0·62 (2·37)	19·92 (137·35)	132	1·99 (3·49)	64·63 (115·61)
Travel patient	286	-	6·07 (30·63)	133	-	61·40 (113·37)
Travel companion	286	-	4·24 (21·34)	133	-	22·61 (44·39)
Hospital stay (days)	286	-	6·09 (88·80)	133	-	0·00 (0·00)
Public	286	0·15 (1·01)	0·34 (3·32)	133	0·07 (0·78)	0·00 (0·00)
Private	286	0·05 (0·61)	5·75 (88·76)	133	0·00 (0·00)	0·00 (0·00)
Total	285	-	38·27 (233·06)	132	-	153·80 (264·21)
**All participants**		**n=720**			**n=360**	
**Baseline**						
Doctor	719	-	50·78 (175·72)	360	-	74·66 (160·46)
Public	720	0·47 (2·16)	5·91 (31·45)	360	0·83 (1·45)	9·71 (37·83)
Private	719	1·18 (3·80)	30·08 (129·75)	360	0·73 (1·67)	33·53 (83·21)
Travel patient	720	-	9·46 (36·35)	360	-	21·43 (57·13)
Travel companion	720	-	5·76 (27·49)	360	-	9·99 (26·78)
Hospital stay (days)	719	-	4·94 (47·56)	360	-	2·10 (16·00)
Public	719	0·11 (0·99)	4·49 (46·95)	360	0·09 (0·66)	1·82 (14·34)
Private	719	0·03 (0·42)	0·45 (7·80)	360	0·01 (0·11)	0·28 (5·35)
Total	718	-	55·80 (184·45)	360	-	76·76 (162·03)
**Month 6**						
Doctor	666	-	24·77 (132·67)	317	-	73·60 (186·61)
Public	667	0·21 (0·79)	3·01 (12·89)	318	0·99 (2·27)	5·25 (11·27)
Private	666	0·33 (1·62)	15·27 (112·70)	317	0·90 (2·47)	31·36 (83·89)
Travel patient	667	-	3·83 (21·31)	318	-	27·03 (78·86)
Travel companion	667	-	2·65 (14·56)	318	-	9·84 (30·71)
Hospital stay (days)	667	-	4·74 (74·30)	318	-	1·53 (23·19)
Public	667	0·09 (0·83)	0·50 (7·37)	318	0·04 (0·55)	0·26 (4·55)
Private	667	0·02 (0·40)	4·24 (73·96)	318	0·19 (3·37)	1·28 (22·75)
Total	666	-	29·52 (190·17)	317	-	75·14 (187·81)

Participants' days off work and lost income

Supplementary table 9 Mean number of days off work and mean lost income at baseline and 6 months, by groups

	mTB-Tobacco			Usual care		
**Lost income or productivity**	**n**	**Mean number of days (SD)**	**Mean lost income (SD), INT$**	**n**	**Mean number of days (SD)**	**Mean lost income (SD), INT$**
**Bangladesh**		**n=400**			**n=200**	
Baseline	400	3·40 (9·45)	75·38 (210·85)	200	5·17 (8·82)	123·90 (255·14)
Month 6	381	3·93 (10·74)	90·87 (256·74)	185	2·90 (6·21)	66·02 (141·67)

Pakistan	n=320			n=160		
Baseline	320	5·07 (18·47)	131·51 (526·32)	160	2·55 (6·89)	52·56 (128·29)
Month 6	286	5·60 (20·00)	155·99 (661·17)	132	4·15 (6·60)	100·16 (174·59)
**All participants**	**n=720**			**n=360**		
Baseline	720	4·14 (14·20)	100·33 (385·17)	360	4·00 (8·12)	92·19 (211·26)
Month 6	667	4·65 (15·41)	118·79 (475·10)	317	3·42 (6·40)	80·23 (156·87)

EQ-5D-5L

Supplementary table 10 Mean EQ-5D-5L utility value and VAS score at baseline and 6 months, by groups

	mTB-Tobacco		Usual care	
	**Baseline**	**Month 6**	**Baseline**	**Month 6**
**Bangladesh**				
n	400	389	200	194
Utility Mean (SD)	0·856 (0·180)	0·944 (0·175)	0·793 (0·226)	0·900 (0·218)
VAS Mean (SD)	60·355 (11·991)	85·077 (14·662)	49·215 (13·503)	81·284 (19·568)
n	389		194	
QALY Mean (SD)	0·454 (0·081)		0·424 (0·105)	
**Pakistan**				
n	320	303	160	151
Utility Mean (SD)	0·691 (0·313)	0·845 (0·287)	0·829 (0·177)	0·836 (0·314)
VAS Mean (SD)	52·575 (19·218)	73·198 (22·268)	59·331 (14·380)	56·033 (27·019)
n	302		151	
QALY Mean (SD)	0·384 (0·131)		0·398 (0·137)	
**All participants**				
n	720	692	360	345
Utility Mean (SD)	0·783 (0·261)	0·900 (0·236)	0·809 (0·206)	0·872 (0·266)
VAS Mean (SD)	56·897 (16·082)	79·876 (19·293)	53·711 (14·764)	70·232 (26·278)
n	691		345	
QALY Mean (SD)	0·423 (0·111)		0·413 (0·121)	

Supplementary table 11 Association of missingness with baseline covariates examined by χ^2 test

	Site	Occupation
**Week 9**		
Costs of sputum tests	**χ^2=51·1472, p=0·002**	χ^2=8·1865, p=0·515
**Month 6**		
Costs of sputum tests	**χ^2=46·1151, p=0·009**	χ^2=7·2544, p=0·611
Costs of TB clinic	**χ^2=46·3508, p=0·008**	**χ^2=21·9944, p=0·009**
Costs of anti-TB medicines	**χ^2=46·1151, p=0·009**	χ^2=7·2544, p=0·611
Costs of doctors & hospital stays	**χ^2=46·3508, p=0·008**	**χ^2=21·9944, p=0·009**
Expenses on TB clinic visits	**χ^2=46·3508, p=0·008**	**χ^2=21·9944, p=0·009**
Expenses on doctor & hospital stays	**χ^2=48·4777, p=0·005**	**χ^2=24·6249, p=0·003**
Expenses on tobacco	**χ^2=238·8802, p=0·000**	**χ^2=38·3271, p=0·000**
Lost income due to sick leave	**χ^2=48·3613, p=0·005**	**χ^2=21·6974, p=0·010**
EQ-5D-5L utility	**χ^2=46·1151, p=0·009**	χ^2=7·2544, p=0·611

χ^2 and p value in bold font denote a significant difference.

Supplementary table 12 Association of missingness with values of the same measure at other data collection points, examined by univariate logistic regression

	Observed values at baseline	Observed values at 6 months
**Missing at week 9**		
Missing costs of sputum tests at week 9	**1·42 (Z=-3·93, p=0·000)**	**1·14 (Z=2·15, p=0·031)**
	**Observed values at baseline**	**Observed values at week 9**
**Missing at 6 months**		
Costs of sputum tests	**1·14 (Z=2·11, p=0·035)**	0·99 (Z=-0·13, p=0·900)
Costs of TB clinic	0·81 (Z=-0·94, p=0·349)	
Costs of anti-TB medicines	No baseline value	
Costs of doctors & hospital stays	1·00 (Z=0·02, p=0·983)	
Expenses on TB clinic visits	1·00 (Z=0·68, p=0·498)	
Expenses on doctor & hospital stays	1·00 (Z=0·08, p=0·934)	
Expenses on tobacco	**1·00 (Z=-2·25, p=0·025)**	
Lost income due to sick leave	1·00 (Z=-0·76, p=0·448)	
EQ-5D-5L utility	1·47 (Z=0·50, p=0·617)	

OR (Z, p) in bold font denote a significant difference.

For analyses conducted on all participants, the number of imputation was set as 13 as the highest percentage of missing data was 13% overall. For country-specific analysis, the number of imputation was set as 6 in Bangladesh and 22 in Pakistan, corresponding to the highest percentage of missing data in the respective countries.

Supplementary material 4 Supplementary results of the primary analysis

Supplementary figure 1 presents cost-effectiveness plane of the incremental cost-utility analysis by country and for all participants.

Supplementary figure 1a shows very little variation, especially in incremental costs, in Bangladesh. This was because the mean costs among non-missing cases concentrated on a few values.

Among non-missing cases, 85% (482/566) did not incur any healthcare costs, 6% (33/566) incurred INT$12·60 healthcare costs per participant, and 7% (42/566) incurred INT$25·20 healthcare costs per participant. Among non-missing cases, 45% (256/566) incurred INT$1·48 TB clinic costs per participant and 25% (142/566) incurred INT$2·22 TB clinic costs per participant. Among those whose routine TB treatment card was available, 97% completed the anti-TB treatment, which meant they incurred same costs of anti-TB medicines. This caused a high number of the bootstrapped replica samples contained a same set of non-missing values even when they drew different participants. Hence, the missing values in each replica sample were imputed with the same values, using predictive mean matching approach. Therefore, it limited the bootstrap variation of estimated incremental costs.

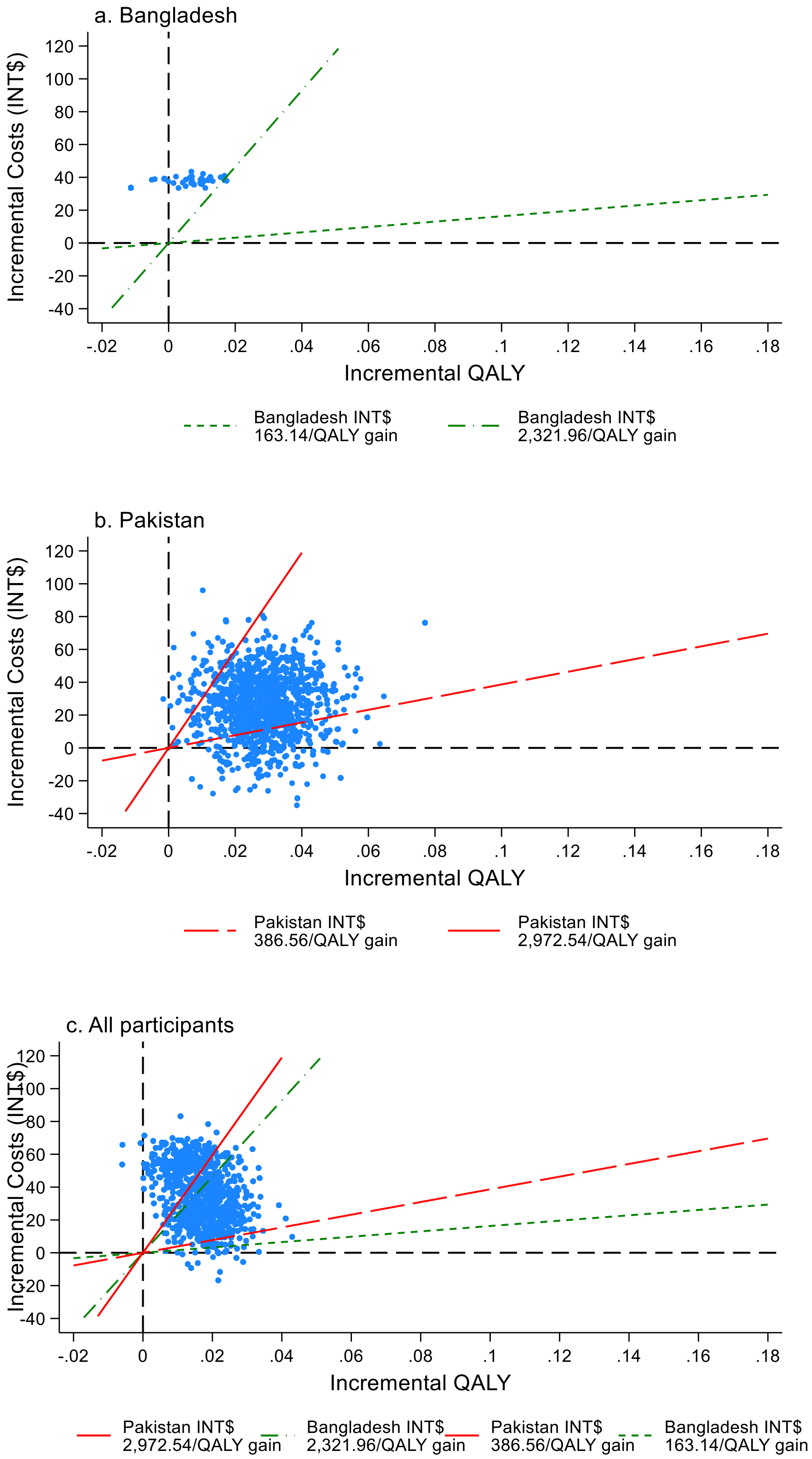

Supplementary figure 1 Sensitivity analysis: Cost-effectiveness planes (CEPs) showing the distribution of bootstrapped incremental costs and QALYs of mTB-Tobacco being cost-effective compared to usual care based on the primary analysis (n=1,080)

Supplementary material 5 Sensitivity analyses: complete case analysis and MNAR examinations

Supplementary table 13 Sensitivity analysis: Complete case analyses of incremental cost-utility analysis, incremental cost-effectiveness analysis for smoking cessation, and incremental analysis of participants' spendings and lost income

	Bangladesh		Pakistan		All participants	
	**mTB-Tobacco (n=380)**	**Usual care (n=185)**	**mTB-Tobacco (n=281)**	**Usual care (n=127)**	**mTB-Tobacco (n=661)**	**Usual care (n=312)**
**Total costs from public healthcare provider perspective, INT$**						
Baseline, mean (SE)	9·29 (1·68)	13·95 (2·58)	53·35 (15·84)	59·15 (15·42)	28·02 (6·85)	32·35 (6·57)
Month 6, mean (SE)	93·99 (1·74)	57·85 (1·39)	140·36 (11·04)	138·43 (15·20)	113·70 (4·88)	90·65 (6·62)
Incremental[a], mean (95%CI)	36·17 (28·29 to 44·27)	-	6·02 (-68·91 to 80·01)	-	21·82 (4·43 to 36·75)	-
**EQ-5D-5L**						
Baseline, mean (SE)	0·859 (0·009)	0·795 (0·017)	0·700 (0·018)	0·836 (0·016)	0·791 (0·010)	0·812 (0·012)
Month 6, mean (SE)	0·964 (0·006)	0·944 (0·007)	0·896 (0·012)	0·951 (0·005)	0·935 (0·006)	0·947 (0·004)
QALY, mean (SE)	0·462 (0·003)	0·442 (0·005)	0·402 (0·007)	0·444 (0·005)	0·436 (0·003)	0·443 (0·004)
Incremental[b], mean (95%CI)	0·002 (-0·008 to 0·013)	-	-0·000 (-0·010 to 0·010)	-	-0·000 (-0·004 to 0·004)	-
Incremental total costs per QALY gained	INT$21,243·57 per QALY gained (Uncertainty see Supplementary figure 2a)	-	More costly, slightly less effective (Uncertainty see Supplementary figure 2b)	-	More costly, slightly less effective (Uncertainty see Supplementary figure 2c)	-
**Incremental treatment costs per abstainer**						
Intervention/control costs, mean (SE), INT$	38·89 (0·00)	1·22 (0·00)	38·65 (0·00)	0·75 (0·00)	38·79 (0·00)	1·03 (0·01)
Biochemically verified continuous abstinence at 6 months (n of abstainer, %)	188, 49·5%	32, 17·3%	108, 38·4%	23, 18·1%	296, 44·8%	55, 17·6%
Incremental treatment costs per one additional abstainer, mean (95% CI)	117·07 (79·07 to 229·01)	-	186·48 (93·30 to 835·87)	-	139·06 (96·81 to 247·66)	-
**Spendings on care, INT$**						
Baseline, mean (SE)	53·00 (8·13)	52·18 (7·60)	80·45 (13·47)	112·60 (18·89)	64·67 (7·40)	76·77 (9·05)
Month 6, mean (SE)	51·27 (7·83)	42·83 (4·81)	39·32 (14·00)	149·84 (23·29)	46·19 (7·46)	86·39 (10·32)
Incremental[c], mean (95%CI)	8·61 (-11·51 to 31·35)	-	-109·13 (-266·63 to 53·68)	-	-43·28 (-59·94 to -24·01)	-
**Lost income, INT$**						
Baseline, mean (SE)	70·49 (10·01)	120·76 (17·69)	147·76 (33·36)	58·25 (15·58)	103·34 (15·37)	95·32 (11·73)
Month 6, mean (SE)	91·10 (13·19)	66·02 (10·42)	154·74 (39·62)	101·28 (15·58)	118·16 (18·49)	80·37 (8·89)
Incremental[d], mean (95%CI)	37·58 (-24·24 to 91·31)	-	98·50 (-108·76 to 328·65)	-	65·15 (11·20 to 137·46)	-

a. For Bangladesh: adjusted for age with site as random intercepts (only one female); For Pakistan: adjusted for age, and sex with site as random intercepts; For all participants: adjusted for age, sex and country with site as random intercepts
b. Adjusted for age, EQ-5D-5L utility at baseline with site as random intercepts
c. By country: adjusted with site as random intercepts; For all participants: adjusted for country with site as random intercepts
d. For Bangladesh: adjusted for occupation, and lost income at baseline with site as random intercepts (only one female); For Pakistan: adjusted for sex, occupation, and lost income at baseline with site as random intercepts; For all participants: adjusted for sex, occupation, country and lost income at baseline with site as random intercepts

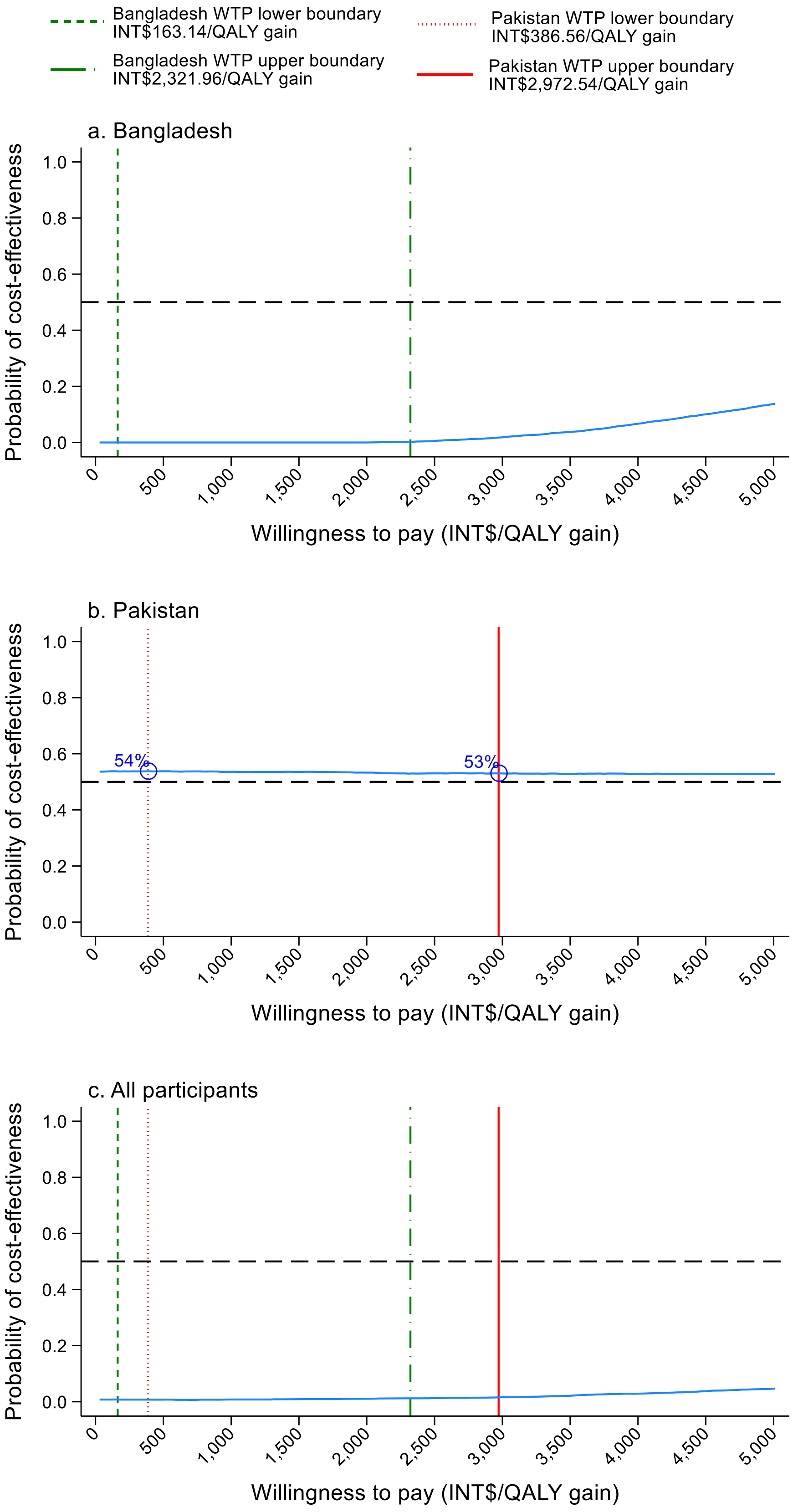

Supplementary figure 2 Sensitivity analysis: Cost-effectiveness acceptability curve (CEAC) showing the probability of mTB-Tobacco being cost-effective compared to usual care based on the complete case analysis (n=973)

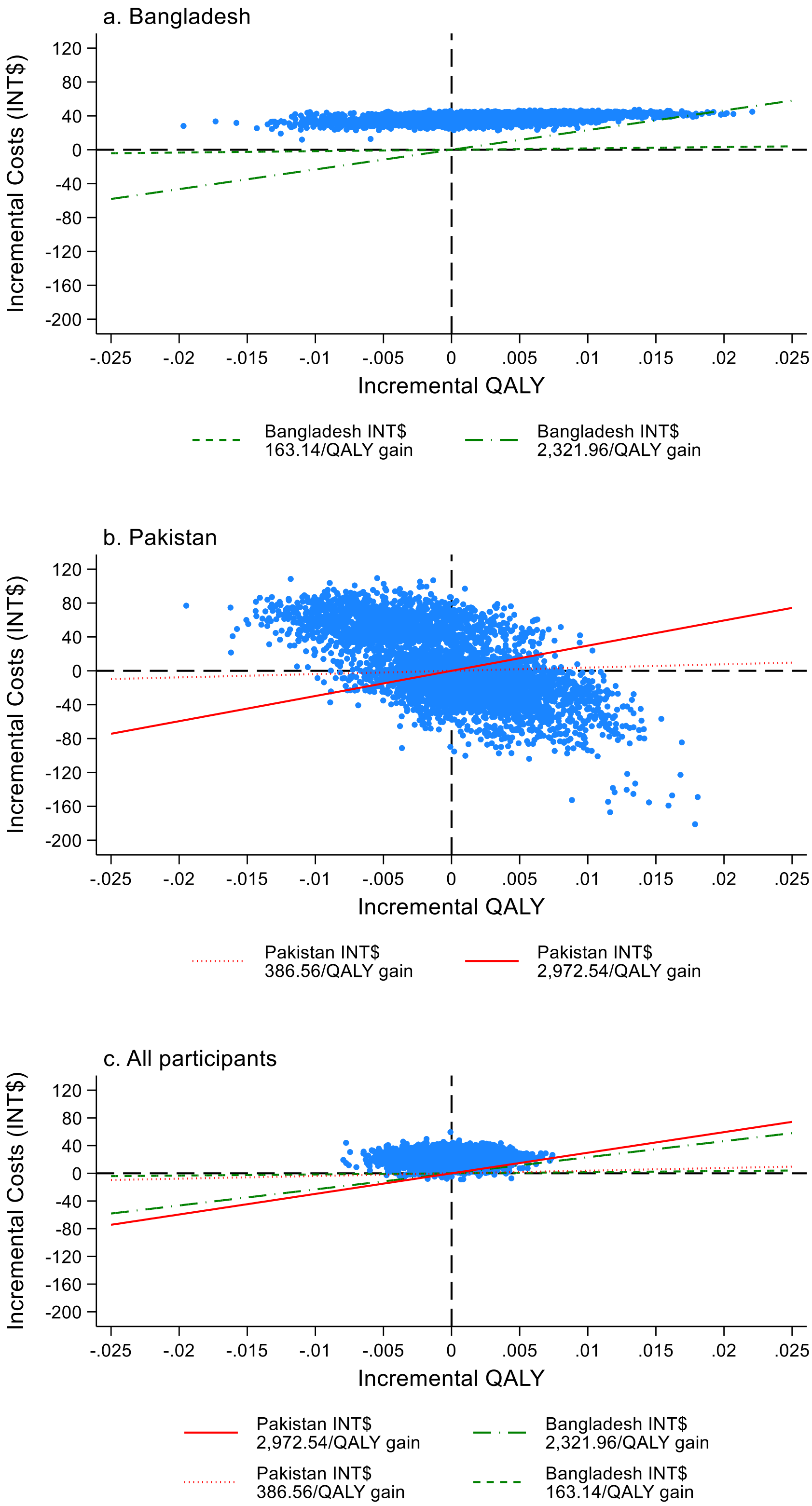

Supplementary figure 3 Sensitivity analysis: Cost-effectiveness planes (CEPs) showing the distribution of bootstrapped incremental costs and QALYs of mTB-Tobacco being cost-effective compared to usual care based on the complete case analysis (n=973)

Supplementary table 14 Sensitivity analysis: Incremental estimates of costs, spendings on care, lost income and QALYs under MNAR scenarios

	Bangladesh (n=600)			Pakistan (n=480)			All participants (n=1,080)		
**Increase imputed costs/OOPs/lost income**									
**INT$**	20%	40%	60%	20%	40%	60%	20%	40%	60%
**Incremental costs[a]**	38·52	38·68	38·84	31·12	33·56	36·01	37·94	39·71	41·48
**Incremental spendings[b]**	6·11	5·61	5·10	-152·44	-162·27	-172·10	-41·60	-42·45	-43·31
**Incremental lost income[c]**	37·49	38·29	39·10	101·34	102·23	103·12	67·38	68·06	68·72
	20%	40%	60%	20%	40%	60%	20%	40%	60%
**Incremental QALYs[d]**	0·008714	0·008853	0·008992	0·028319	0·028467	0·028612	0·016889	0·017053	0·017217
ICER									
**Minimum**	20% increase of imputed costs with 60% reduction of imputed utility: INT$4,283·90 per QALY gained			20% increase of imputed costs with 60% reduction of imputed utility: INT$1,087·67 per QALY gained			20% increase of imputed costs with 60% reduction of imputed utility: INT$2,203·69 per QALY gained		
**Maximum**	60% increase of imputed costs with 20% reduction of imputed utility: INT$4,457·09 per QALY gained			60% increase of imputed costs with 20% reduction of imputed utility: INT$1,271·58 per QALY gained			60% increase of imputed costs with 20% reduction of imputed utility: INT$2,456·01 per QALY gained		

a. For Bangladesh: adjusted for age with site as random intercepts (only one female); For Pakistan: adjusted for age, and sex with site as random intercepts; For all participants: adjusted for age, sex and country with site as random intercepts
b. By country: adjusted with site as random intercepts; For all participants: adjusted for country with site as random intercepts
c. For Bangladesh: adjusted for occupation, and lost income at baseline with site as random intercepts (only one female); For Pakistan: adjusted for sex, occupation, and lost income at baseline with site as random intercepts; For all participants: adjusted for sex, occupation, country and lost income at baseline with site as random intercepts
d. Adjusted for age, EQ-5D-5L utility at baseline with site as random intercepts
